# Enactivism is not interactionism

**DOI:** 10.3389/fnhum.2012.00345

**Published:** 2013-01-03

**Authors:** Hanne De Jaegher, Ezequiel Di Paolo

**Affiliations:** ^1^Department of Logic and Philosophy of Science, IAS-Research Centre for Life, Mind, and Society, University of the Basque CountrySan Sebastián, Spain; ^2^Department of Informatics, Centre for Computational Neuroscience and Robotics, and Centre for Research in Cognitive Science, University of SussexBrighton, UK; ^3^Ikerbasque, Basque Foundation for ScienceBilbao, Spain

**A commentary on**

**Toward an integrative account of social cognition: marrying theory of mind and interactionism to study the interplay of Type 1 and Type 2 processes**

by Bohl, V., and van den Bos, W. (2012). Front. Hum. Neurosci. 6:274. doi: 10.3389/fnhum.2012.00274

Bohl and van den Bos (2012) sketch an approach to the study of social cognition aimed at integrating mindreading and alternatives based on social interaction. Inspired by dual-process models, they draw an analogy between, on the one hand, Type 1 processes (fast, automatic, and situated) and processes involved in supporting interaction and, on the other, Type 2 processes (slow, volitional, and domain-general) and mindreading. The proposal has empirical potential, as interactive factors begin to be more systematically investigated in neuroscience.

Here, we clarify that what the authors describe as “interactionism”—and attribute in its radical form to us—is, in important ways, different from the enactive approach we defend. We argue for the *necessity* of including interactive factors in at least *some* forms of social understanding, and, as a consequence, for the *insufficiency* of mindreading to account for *all* of social cognition. But the authors misinterpret our position. They suggest that it implies the *sufficiency* of interactive factors for explaining *all* of social understanding and, therefore, the *non-necessity* of mindreading in *any* particular case of social cognition. Overlooking the caveat “*in some cases*” in this quote “[…] we can conceive of interaction dynamics as, in some cases, delivering the necessary cognitive performance” (De Jaegher et al., 2010), they interpret that “individual explanations become superfluous once the interaction process is explained on the supra-individual level” *without* the caveat (p. 4). In effect, they make universal a claim about particulars. In our view, they suggest, everything reduces to a monopolizing strategy (understanding interactions) and individual factors, including reasoning about others, are only secondary.

This misperception may result from a “contrast effect” whereby after staring into the dark for too long new shades of gray are initially seen as blinding white.

In dynamical systems terms, the coupling between two systems is constrained by internal processes in each of them. *Social interaction* is a coupling between two or more autonomous agents that is co-regulated by the interactors (they modify their coupling to satisfy some condition; e.g., approaching a speaker in the presence of loud ambient noise so as to hear them better) and the resulting relational dynamics acquires a form of autonomy (De Jaegher et al., 2010, p. 493). Interactions depend on individual contributions, but are not fully determined by them. They depend also on the relational dynamics between subjects, and other factors. According to our definition, studying interaction requires an understanding of the relation between the *individual* and *collective* levels. This is why we criticize sociological analyses of interaction for not paying enough attention to individual cognition (De Jaegher and Di Paolo, 2007, p. 492).

The enactive approach makes two main moves; first, it posits systemic concepts for understanding social interactions. Second, it examines how interaction affects sense-making, i.e., how intentional phenomena are modulated by patterns of coordination, breakdowns, and recoveries between interactors. This is participatory sense-making.

Does this mean that individual sense-making, including abilities that could be described as mindreading, cannot occur? No. We never suggested that individual cognitive performances are not relevant to some forms of social cognition. In fact, we explicitly call for a reconsideration of individual mechanisms (De Jaegher et al., 2010, p. 445). In our example of interaction over a delayed communication line misunderstanding arises from a combination of interactive and interpretive factors (De Jaegher and Di Paolo, 2007, p. 498). To be sure, we analyze in detail situations where interactions are central and abstract reasoning less so (Murray and Trevarthen, 1985; Auvray et al., 2009). Our argument would be weak if we couldn't show empirical instantiations of our claim.

Only in a recent paper do we move into more radical terrain (Di Paolo and De Jaegher, 2012). The Interactive Brain Hypothesis states that the brain processes at play during social cognition are functionally shaped by interactions (among other things!) or that their functionality co-opts that of individual processes at play during interactions. This is indeed more radical, but it is a hypothesis open to empirical refutation. And even this is still not the same as saying that only interaction matters.

Bohl and van den Bos state that enactivism focuses on cooperative, smooth interactions. But our claims do not depend on this. In fact, participatory sense-making relies crucially on coordination breakdowns. Breakdowns and recoveries are basic to the generation of novel social meaning. Without at least a minimal element of conflict there would not be social understanding. We discuss antagonistic interactions involving misunderstandings (e.g., De Jaegher and Di Paolo, 2007, p. 498) and consider conflict and interactive escalation (Di Paolo and De Jaegher, 2012, p. 7, 10).

The authors complain that the enactive perspective underplays subpersonal processes. However, we use dynamical models to explain experimental results (e.g., Di Paolo et al., 2008; Froese and Di Paolo, 2010). Like the explanation we propose for perceptual crossing or the double TV-monitor experiments, these are strictly subpersonal and span individual and transindividual factors. Phenomena at this level of analysis (fields, attractors, transients, etc.) do not involve intentional-level terminology, unlike so-called “subpersonal” processes proposed in mindreading explanations (“simulations” and “inferences”). In contrast to functionalism, enaction takes the subpersonal level very seriously and avoids mereological fallacies and homuncular explanations that nullify the very idea of a subpersonal level.

With these clarifications, we see Bohl and van den Bos's proposal as a research heuristic that can surely enrich empirical data. However, we worry about whether this is a long-term integrative approach.

There are two ways of understanding the proposal:

1. Individual sense-making is largely supported by Type 2 processes and interaction by Type 1 processes.
2. The *relation* between individual sense-making and interactive performances is analogous to the *relation* between Type 2 and Type 1 processes.

The first reading is problematic for those who claim that implicit mindreading is supported by automatic processes that do not involve volitional control (Type 1), as noted by the authors (p. 8). And deliberative Type 2 processes that are not involved in the performance of mindreading can still occur in interactions: for instance, when two people try to solve a maths problem together. Such processes can constrain the interaction dynamics and influence how interaction affects mutual understanding.

We therefore understand the proposal as an analogy (case 2) that calls for similar methodologies as those used in researching Type1/Type2 processes.

However, the proposal must not amount to an uncritical combination of two approaches. A theory of intersubjectivity should address issues like: what are the underlying principles that relate skilful interaction and individual sense-making of others? What neural/bodily mechanisms are involved in each or indeed shared? Is there a developmental “flow” between skilful interactive and individual sense-making capabilities? Is it a two-way flow? How much do the two “types” interpenetrate, not just developmentally but in the enaction of social understanding? Can they always (ever?) be described as distinct in principled ways?

Like other hybrid approaches in cognitive science and biology (gene-environment, nature-nurture, symbolic-connectionist, etc.), the proposal must avoid certain pitfalls. One is the risk of reifying the descriptive elements (Types 1 and 2). Another is to take the distinction between them as clear-cut, foregoing considerations of how they interpenetrate. A hybrid approach can also lead to explanations based on “contributions” (“a performance is 80% interactive and 20% mindreading”). This strategy is weak in the long term. And in general, there is a risk of adding epi-cycles when arguably what the field needs is a Copernican shift.

We applaud the authors for recognizing interaction as important. But we don't think dichotomous frameworks can achieve long-term theoretical integration. Hence our clarifications: enactivists already do not think that all that matters happens only in interaction. We criticize methodological individualism but do *not* thereby hold true its exact opposite (the irrelevance of individual cognition). The enactive stance attempts to supersede such a dichotomy. In that sense, the motivations of the authors are aligned with those of enactivism.
